# An Improved RAPID Imaging Method of Defects in Composite Plate Based on Feature Identification by Machine Learning

**DOI:** 10.3390/s22218413

**Published:** 2022-11-01

**Authors:** Fei Deng, Xiran Zhang, Ning Yu, Lin Zhao

**Affiliations:** School of Electrical and Electronic Engineering, Shanghai Institute of Technology, Shanghai 200235, China

**Keywords:** lamb wave, composite plate, nondestructive testing, defect imaging, machine learning

## Abstract

The RAPID (reconstruction algorithm for probabilistic inspection of defect) method based on Lamb wave detection is an effective method to give the position information of a defect in composite plate. In this paper, an improved RAPID imaging method based on machine learning (ML) is proposed to precisely visualize the location and features of defects in composite plate. First, the specific feature information of the defect, such as type, size and direction, can be identified by analyzing the detection signals through multiple machine learning models. Then, according to the obtained defect features, the scaling parameter *β* of the RAPID method which controls the size of the elliptical area is revised, and weights are set to the important detection paths which are related to defect features to realize precise defect imaging. The simulation results show that the proposed method can intuitively characterize the location and related feature information of the defect, and effectively improve the accuracy of defect imaging.

## 1. Introduction

Composite materials with fatigue resistance, light weight, and high strength are widely utilized in aerospace, construction, and other fields [1,2]. Surface or internal defects such as delamination, crack, debonding, pore, inclusion, looseness, and rich resin may occur in the manufacturing and service process of composite materials [3]. Lamb wave-based detection has been widely used in the detection of composite materials due to its long propagation distance, small attenuation, and sensitivity to small defects [4,5]. The anisotropy of the composite plate results in the change of the propagation behavior of guided waves in the plate structure [6], and the complex transformation of waveform aggravates the complexity of interpreting the detection signal [7,8]. At the same time, the complexity of the detection signal also increases the difficulty of precise defect imaging in the composite plate.

Many damage imaging methods can be used for defect detection in composite plates, such as the ellipse imaging method [9,10], delay-and-sum imaging method [11,12], time-reversal imaging method [13,14], RAPID imaging method [15,16,17,18], etc. The RAPID can intuitively present the location of defects by analyzing the statistical characteristics of the detection signal, and has been widely studied by researchers. Wang et al. [19] studied the mode of directional scattering of cracks in aluminum plate and proposed an improved probabilistic damage imaging algorithm, which achieved defect imaging by finding the path with the smallest signal difference coefficient to predict the crack direction. Wu et al. [20] studied the influence mechanism of several parameters in the probabilistic damage imaging method, and proposed a method to optimize the sensor network and determine these parameters, which was verified on the composite stiffened panel. In the above studies of probabilistic damage imaging methods, the specific characteristics of defects (e.g., length and direction of cracks, size of delaminated areas) cannot be precisely known, which reduces the accuracy of defect imaging and affects the application of imaging methods in practice.

The combination of Lamb wave detection and ML can be used to identify defects. In general, according to prior knowledge in the field, damage-sensitive features are analyzed and extracted as input training model through a variety of signal processing methods to realize the identification of damage types and degrees [21]. Zhou et al. [22] extracted the relevant characteristics of the time domain, the frequency domain, and the energy of the Lamb wave signal as input to the classifier, and studied the degree of corrosion damage at the bend of the pipeline. Experimental results show that machine learning can be used to identify the damage category of defects, and support vector machine (SVM) has a better identification effect under the condition of small samples. Peng et al. [23] estimated the size and location of the defect by obtaining the damage-sensitive features of the guided wave signal and incorporating them into a Bayesian update framework. Mardanshahia et al. [24] extracted different features of Lamb wave signal to train three supervised ML methods to classify the density of cracks. However, for anisotropic composite plates, the propagation law of Lamb waves is more complicated, and it relies too much on existing prior knowledge to extract features and cannot perform damage detection highly efficiently.

In this paper, a defect imaging method based on an improved RAPID algorithm is proposed for defects in composite plates. Before the defect detection, the guided wave detection baseline signal is obtained by a circular sensor array arranged in the defect-free composite plate through simulation or experiment. Then defects with different types and parameters are pre-constructed in the center of the array to obtain the corresponding damage detection signal for constructing the defect feature identification sample library. And based on these defect detection signals, the ML models for defect feature identification are trained, respectively. For the composite plate to be inspected, the above-mentioned circular sensor array is first used to obtain the detection signals of the inspected area. Then, based on these detection signals and the reference signals, the DI values of each path are calculated, and path selection is performed based on these DI values. For the selected paths, pre-location is performed using RAPID to obtain the center coordinates of the defect. After that, the center of the sensor array is moved to the predicted defect location to obtain the detection signals for feature recognition. On this basis, the corresponding models are used to identify the defect types and main geometric parameters (such as the length and direction of cracks, the size of the delamination area, etc.). Finally, after obtaining the characteristic parameters of the defects, the RAPID algorithm is improved by modifying the *β* values and setting the weights of the relevant detection paths to refine the imaging of different types of defects. Based on theoretical analysis, this paper takes two types of defects, notably crack and delamination, in composite plates as examples to carry out simulation experiments and research to verify the effectiveness of the above methods. The simulation results show that the proposed method can intuitively characterize the location and related feature information of the defect, and effectively improve the accuracy of defect imaging.

The abbreviations involved in the proposed method and their meanings are shown in Table 1.

## 2. Methods

The RAPID is based on the correlation coefficient of the reference and damage signals, and the location relationship among the transmitter, the receiver, and the defect. It avoids the complex process analysis of Lamb wave propagation to achieve defect localization. The proposed method fuses the defect feature predicted by the ML method into the RAPID algorithm to achieve precise defect imaging.

The mode conversion, reflection, and transmission process of the Lamb wave is affected by the characteristics of the defect which include type, size, location, and other factors. Such influence causes the detection signal to change. The closer the defect is to the sensing path, the more influence it has on Lamb wave signal. Conversely, the farther the relative distance is, the smaller the influence on the Lamb wave signal. Therefore, RAPID imaging methods can roughly estimate the location of defects.

### 2.1. Path Selection and Defect Pre-Locating

**Figure 1:**
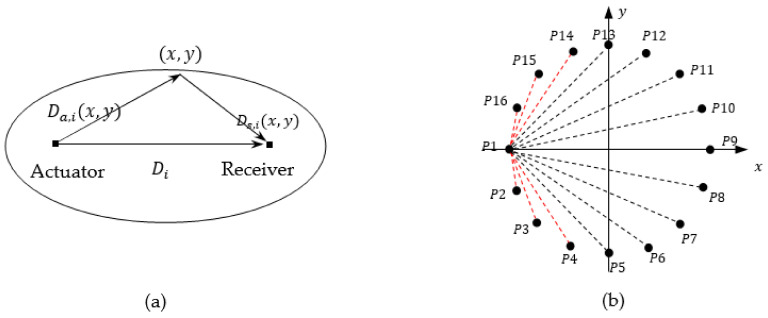
Schematic diagram of RDI and path selection: (**a**) Schematic diagram of $RD_{i}$; (**b**) Schematic diagram of the path selection.

In the process of using the RAPID, the composite plate is divided into uniform grids, and each grid corresponds to a pixel. For each pixel, its value is the probability value of existence of damage. The probability of the presence of the defect at position (*x*, *y*) can be defined as:

(1) $$P(x,y)=\sum_{i=1}^{N}{DI}_{i}\cdot \frac{\beta -R_{i}(x,y)}{\beta -1}$$

where *N* is the number of participating sensing paths, $\left[\beta -R_{i}\left(x,y\right)\right]/\left(\;\beta -1\right)$ is the spatial distribution function of the ith path, and its outline is an ellipse. The parameter β is a scaling parameter that controls the size of the ellipse distribution area. It is usually set around 1.0, which is an empirical value determined on a case-by-case basis, but such treatment is not always suitable for all situations in practice. In this paper, the pre-locating process sets β=1.05  [25]. The term $R_{i}\left(x,y\right)$ is defined as follows:

(2) $$R_{i}(x,y)=\left\{\begin{array}{c} RD_{i}(x,y),RD_{i}(x,y)<\beta \\ \beta ,RD_{i}(x,y)\geq \beta \end{array}\right.$$

where $RD_{i}\left(x,y\right)=\left[D_{a,i}\left(x,y\right)+D_{s,i}\left(x,y\right)\right]/D_{i}$,$D_{a,i}\left(x,y\right)$,$D_{s,i}\left(x,y\right)$ is the distance from the point $\left(x,y\right)$ to the excitation point and the receiving point, respectively. $D_{i}$ is the distance from the excitation point to the receiving point, as shown in Figure 1a.

$DI_{i}$ is the damage factor of the ith path, defined as follows:

(3) $$\begin{array}{cc} DI=1-\rho & =1-\frac{\sum_{k=1}^{n}\left(X_{k}-\bar{X_{k}})(Y_{k}-\bar{Y_{k}}\right)}{\sqrt{\sum_{k=1}^{n}{\left(X_{k}-\bar{X_{k}}\right)}^{2}{\left(Y_{k}-\bar{Y_{k}}\right)}^{2}}}\\ & =1-\frac{n\sum_{k=1}^{n}X_{k}Y_{k}-\sum_{k=1}^{n}X_{k}\cdot \sum_{k=1}^{n}Y_{k}}{\sqrt{\left[n\sum_{k=1}^{n}X_{k}^{2}-{\left(\sum_{k=1}^{n}X_{k}\right)}^{2}][n\sum_{k=1}^{n}Y_{k}^{2}-{\left(\sum_{k=1}^{n}Y_{k}\right)}^{2}\right]}} \end{array}$$

where ρ is the correlation coefficient between the baseline data and the detection data. $X_{K}$ and $Y_{k}$ are the baseline signal and inspection signal, respectively. ${\bar{X}}_{k}$ and ${\bar{Y}}_{k}$ are the mean of $X_{K}$ and $Y_{K}$, respectively. *k* is the number of recorded points in the data.

The magnitude of the DI value reflects the degree of correlation between the sensing path and the defect. The rule for path selection is as follows: using P1 as the excitation sensor and the rest of the sensors as the receiving sensors as an example, the circular array is shown in Figure 1b. A coordinate system with path P1–P9 as the *x*-axis and path P5-P13 as the vertical *y*-axis is created. The sensing paths in the second and third quadrants are ignored, and the remaining paths are the filtered paths. The DI values of these remaining paths are calculated according to Equation (3), then the larger two are selected as valid *DI* values. On this basis, the final *DI* values is obtained by filtering again through considering the reciprocity of the paths.

### 2.2. Defect Classification and Feature Identification

Under the same detection conditions, the detection signals generated by the same type of defect have some common characteristics, and this lays a foundation for the use of ML methods to realize defect feature recognition.

SVM is a ML method based on statistical theory. It maximizes the interval between classes by finding a “hyperplane”. Additionally, the kernel function is utilized to map the data from a low-dimensional space to a high-dimensional space to make the data linearly separable. SVM has great advantages for data with high dimensions, small sample size, and nonlinearity [26,27]. It can be used for the identification of defect types and defect sizes.

Light GBM [28] is a distributed gradient lifting framework with the decision tree as a weak learner. It innovatively uses the gradient-based unilateral sampling algorithm and mutually exclusive feature binding algorithm to conduct data sampling and feature sampling, respectively. In addition, it does not need to traverse all the data in each iteration and can improve the training speed with the same accuracy. Although the model has high complexity, it is very suitable for classification tasks with high dimension. The model is used to identify crack direction in this study.

### 2.3. RAPID Algorithm for Fusing Defect Features

**Figure 2:**
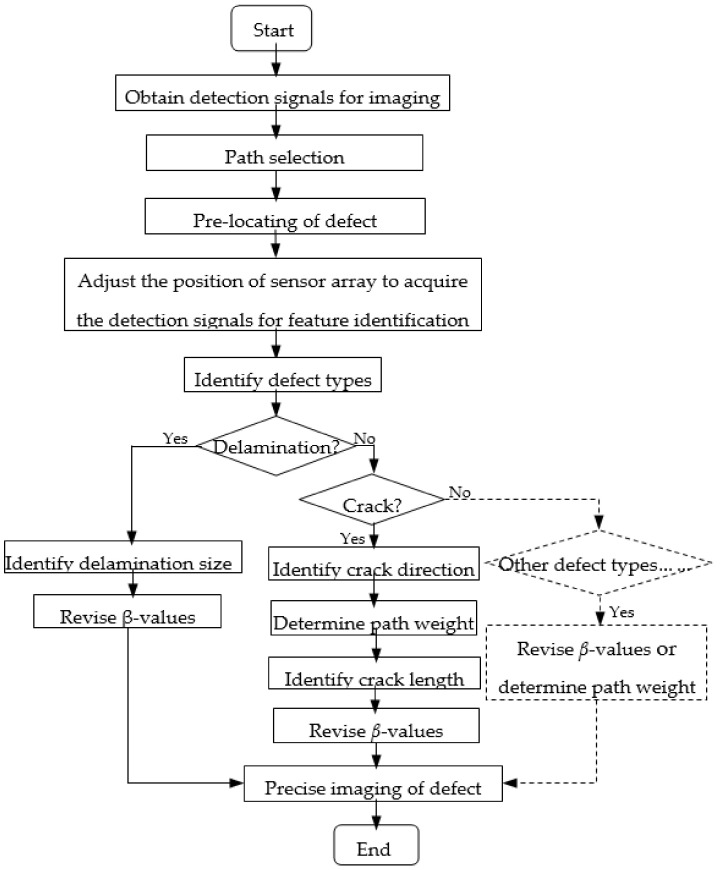
The flow diagram of the proposed method.

Sheen et al. [29] proposed that the *β* value can be further adjusted to achieve precise imaging based on knowing the size of the defect. Therefore, according to the main characteristics (type, size, orientation) of the defect obtained by the ML method, the parameter β is precisely corrected to improve the quality of the defect imaging. Figure 2 is a flow chart of the improved imaging algorithm.

**Figure 3:**
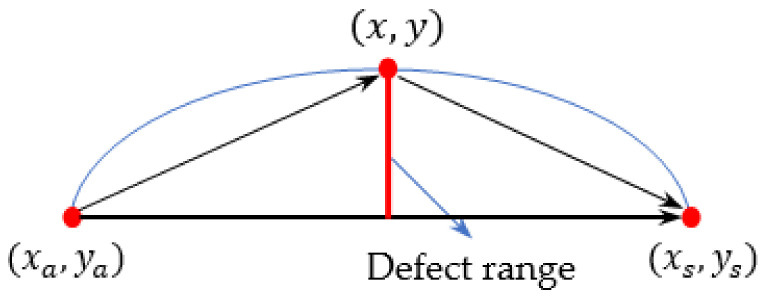
Schematic diagram of the calculation of *β*.

The calculation of *β* is shown in Figure 3, where the defect range is described as the maximum vertical distance between the defect boundary and the detection path. In this paper, two kinds of defects (delamination and cracks) are studied as follows.

#### 2.3.1. Delamination

In the detection path, the sum of the distances from the farthest point of the delamination defect to the excitation and receiver is calculated first, then the ratio of this sum value and the distance between excitation and receiver can be used to revise the β value as follows:

(4) $$\beta =R_{Max\_defect\_range}=\frac{\sqrt{{\left(x_{a}-x\right)}^{2}+{\left(y_{a}-y\right)}^{2}}+\sqrt{{\left(x_{s}-x\right)}^{2}+{\left(y_{s}-y\right)}^{2}}}{\sqrt{{\left(x_{a}-x_{s}\right)}^{2}+{\left(y_{a}-y_{s}\right)}^{2}}}$$

Here, $\left(x_{a},y_{a}\right)$,$\;\left(x_{s},y_{s}\right)$, and $\left(x,y\right)$ are the coordinates of the excitation, receiver, and of the point which is farthest from the sensing path on the delamination boundary, respectively.

#### 2.3.2. Crack

**Figure 4:**
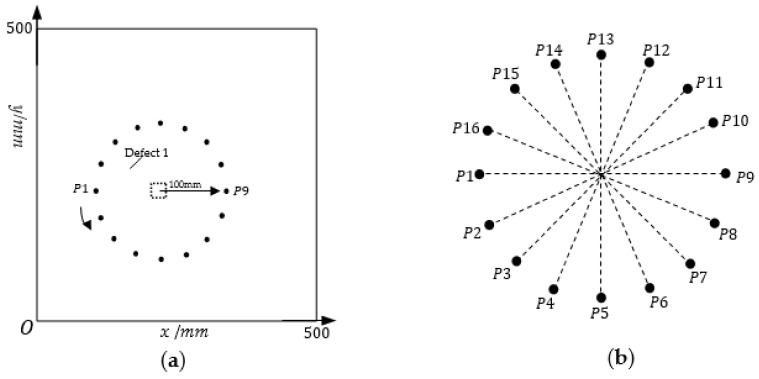
Schematic diagram of the experimental setup: (**a**) sensor arrangement and defect setting; (**b**) sensing path.

The width of the crack is extremely narrow. Therefore, if the direction of path *i* is consistent with the direction of crack (defined as the angle between the crack and the positive *x*-axis in the coordinate system shown in Figure 4a), it can be empirically set to $\beta_{0}$ = 1.0001. After the length value of the crack is predicted by the ML model, half of the length value is considered to be the defect range. On the basis of obtaining the defect range, and knowing $\left(x_{a},y_{a}\right)$ and $\left(x_{s},y_{s}\right)$, the β can be adjusted according to the following formula:

(5) $$\beta =\left\{\begin{array}{ll} RMax\_defect\_range, & \;\mathrm{the}\;\mathrm{direction}\;\mathrm{of}\;\mathrm{path}\;i\;\mathrm{is}\;\mathrm{not}\;\mathrm{consistent}\;\mathrm{with}\;\mathrm{the}\;\mathrm{crack}\;\mathrm{direction}\\ \beta_{0}, & \;\mathrm{the}\;\mathrm{direction}\;\mathrm{of}\;\mathrm{path}\;i\;\mathrm{is}\;\mathrm{consistent}\;\mathrm{with}\;\mathrm{the}\;\;\mathrm{crack}\;\mathrm{direction}\; \end{array}\right.$$

In addition, the path closest to the crack direction and the path perpendicular to the crack direction form the “cross path”. The weight $W_{i}$ of the above two paths should be set much larger than the other paths, and its weight can be set as:

(6) $$W_{i}=\left\{\begin{array}{l} \frac{L}{l},\;\mathrm{cross}\;\mathrm{paths}\\ 1\;,\;\mathrm{other}\;\mathrm{paths} \end{array}\right.$$

where $L\left(mm\right)$ is the diameter of the circular sensing array; and $l\left(mm\right)$ is the crack length. The smaller the crack length is, the greater the weight applied.

In summary, for the crack, the modified damage imaging algorithm is defined as:

(7) $$P(x,y)=\sum_{i=1}^{N}{DI}_{i}\cdot \frac{\beta -R_{i}(x,y)}{\beta -1}\cdot W_{i}$$

In order to illustrate the proposed method more specifically, the pseudocode of the improved methods is given in Algorithms 1–3. Algorithms 1 and 2: the pseudocode for feature recognition. Algorithm 3: the main pseudocode of crack in the proposed method.
**Algorithm 1:** Train the SVM model**Input:***D*: training set
*D*1←StandardScaler (*D*)
*D*2←Dimension reduction: PCA (*D*1)

Params←[{C}, {kernel function}]

C, kernel function←GridSearchCV(SVM, params)
**return** the SVM model

**Algorithm 2:** Feature identification by ML models**Input:***D*1: detection signal acquired at paths P1–P9 after adjusting the position of the   sensing array

   *D*2*:* detection signals acquired in paths P1–P9, P2–P10, P3–P11, P4–P12, P5–P13,

   P6–P14, P7–P15, P8–P16 after adjusting the position of the sensing array
**Input:***I*_1_: the model for identifying the defect type

   *I*_2_: the model for identifying the delamination size

   *I*_3_: the model for identifying the crack direction

   *I*_4_: the model for identifying the crack size

preds←*I*_1_. predict (*D*1)
**if** preds==delamination **then**

| the size of delamination←*I*_2_. predict (*D*1)
**else if** preds==crack **then**

| *f* (the direction of crack)←*I*_3_. predict (*D*2), *l* (the size of crack)←*I*_4_. predict (*D*1)

**else**

|……

**end**
**return** the features of the defect

**Algorithm 3:** Precise imaging of crack

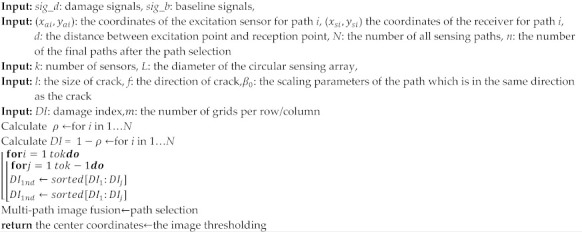



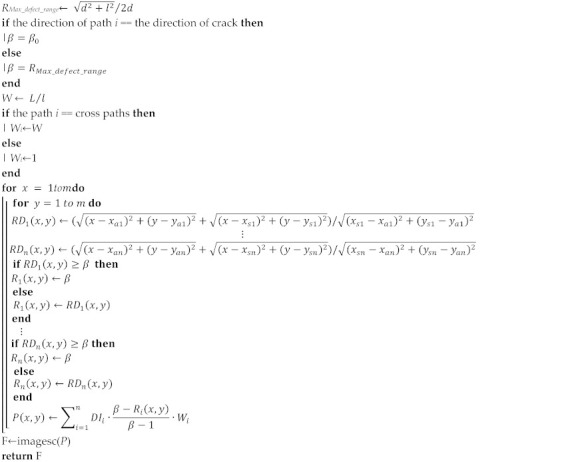



## 3. Experimental Setup

A three-dimensional finite element analysis is performed with ABAQUS software. A [45°/−45°/0°/90°] quasi-isotropic composite laminate with a dimension of 500 mm × 500 mm × 1.28 mm is considered in the coordinate system shown in Figure 4a. The material properties of the laminate are listed in Table 2. A circular sensor array (radius r = 100 mm) composed of 16 evenly distributed sensors is arranged on the surface of the laminate. The excitation signal used in the numerical study is a 160-kHz narrow-band five-cycle sinusoidal tone burst modulated by a Hanning window. Loading is performed at each sensor position in turn, and the received signal is obtained at the remaining sensor positions. A total duration of 300 μs time length is solved using ABAQUS/Explicit dynamical analysis with a fixed step size of 10^−8^ s for all the numerical cases.

### 3.1. Building the Sample Library

This study only discusses two typical types of defects in the composite plate: delamination and cracks. On the defect-free simulation model mentioned above, the delamination or crack defect with changing geometric parameters is set at the center of the circular sensor array which is shown in Figure 4b, and the detection signals are acquired by one-excitation, one-receive pattern to construct a sample library.

As shown in Figure 4a, a rectangle of variable length and width corresponding to the dashed box is used to simulate the delamination defects. Due to computational limitations, the obtained samples do not cover all delamination sizes. In the sample library for this simulation experiment, only the following nine classes are considered: 5 mm × 5 mm, 10 mm × 10 mm, 15 mm × 15 mm, 5 mm × 10 mm, 5 mm × 15 mm, 10 mm × 5 mm, 10 mm × 15 mm, 15 mm × 5 mm, and 15 mm × 10 mm, and the corresponding sample labels are set as 1, 2, 3, 4, 5, 6, 7, 8, and 9, respectively, as shown in Table 3. For the composite plate studied in this paper, the delamination defects are set between the top layer and the adjacent second layer, and between the second layer and the adjacent third layer, respectively. The variations of the length and width of the defects are shown in Table 3. Classes 1, 2 and 3 contain 84 samples, respectively, and classes 4, 5, 6, 7, 8 and 9 contain 44 samples, each. In addition, in order to increase the robustness of the model, samples that are between classes are also supplemented in the experiments, such as adding samples of 5 mm × 8 mm, 5 mm × 14 mm, 10 mm × 6 mm, 10 mm × 16 mm, 15 mm × 6 mm, and 15 mm × 11 mm. Although the length and width directions of the rectangles can be changed, this paper does not consider such changes for the time being and only assumes that the length and width directions of the rectangles are the same as the length and width directions of the plates. Since in the method proposed in this paper, the detection signal is closely related to the variation of defects in the direction perpendicular to the direction in which the path is located, only the detection signal acquired on the path P1-P9 as shown in Figure 4b is selected here for the parametric classification identification of layered defects.

The main geometrical features of the crack include length and direction. For the direction of the crack, there are 7 categories: 0°, 30°, 45°, 60°, 90°, 120°, 135°. The ply direction of composite plate and the crack direction itself will comprehensively affect the detection signal, so the detection signals on the detection paths which is in different directions need to be acquired for direction identification. The detection paths include path P1–P9, P2–P10, P3–P11, P4–P12, P5–P13, P6–P14, P7–P15, and P8–P16 as shown in Figure 4b. For the same crack direction, the crack length is set to 3 mm, 4 mm, ..., 12 mm, respectively. In the same crack length category, the length is varied in 0.01mm steps to obtain 88 samples of defects with length differences. For example, 2.68 mm, 2.69 mm, ......, 3 mm, 3.01 mm, ......, 3.55 mm is considered to be a variation of the length of the 3 mm class. Thus, 880 samples are included under each direction class, and a total of 6160 samples are obtained.

### 3.2. Building the Testing Defects

To illustrate the implementation process of the optimized RAPID imaging algorithm, three representative defects are selected as test samples: defect 1, an oblique crack with a length of 12.05 mm and an angle of 45° is set on the surface of the composite plate, and its coordinates are$\;\left(x,y\right)=(186,264$); defect 2, a vertical crack with a length of 4.1 mm and an angle of 90° is set on the surface of the composite plate, and its coordinates are$\;\left(x,y\right)=(263,225$); defect 3, a rectangle shape representative delamination with the size of 5 mm × 5.2 mm is modeled at the coordinates of the composite plate $\left(x,y\right)=\left(225,263\right)$ between the first layer (top surface) and the adjacent second layer of the composite plate. The sensor array and the setting of defect 1 are shown in Figure 4a.

## 4. Identification Model

### 4.1. Identification Model of Defect Type

**Figure 5:**
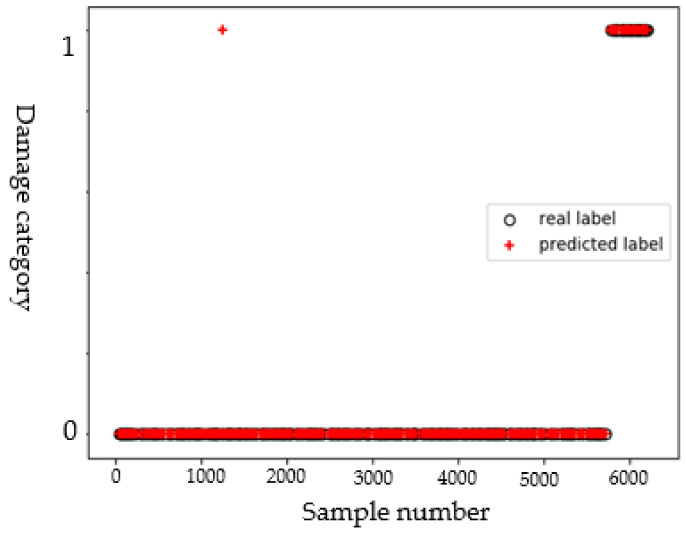
Scatter diagram of identification results of the defect type.

The effect of delamination and cracks on the propagation behavior of guided waves is different. On the detection path passing through the center of the circle, the signal is obtained by one-excitation and one-receive pattern for building a sample library. According to exciting frequency and sampling frequency, each detection signal contains 30,000 data points, and the signal is taken as a 30,000-dimensional sample. The sample library of defect type is constituted by 522 detection signals of delamination and 6160 detection signals of the crack. It is randomly divided into training set and test set with a ratio of 8:2. Because the dimension of the samples is too high, the Principal Component Analysis (PCA) method [30,31] is utilized for pre-processing. Samples of the training set are used as input of the SVM classifier, and the label set is formed by the defect classes of the corresponding samples (0 and 1 represent crack and delamination, respectively). The grid optimization method and cross-validation are used to adjust the kernel function type and the main model parameters. The kernel function is set as the linear kernel function, and the penalty factor (*C*) is 3. The average accuracy rate, the recall rate, and $F_{1}$ score obtained from 10 experiments are about 96.74%, 97.81%, and 96.04%, respectively. It can be seen from the scatter plot in Figure 5 that most of the samples are correctly classified and only one sample from the crack class is misclassified into the delamination class. Crack and delamination have different effects on the propagation behavior of Lamb waves, which enables the model to better identify the two types of defects.

### 4.2. Parameter Identification Model for Delamination

**Figure 6:**
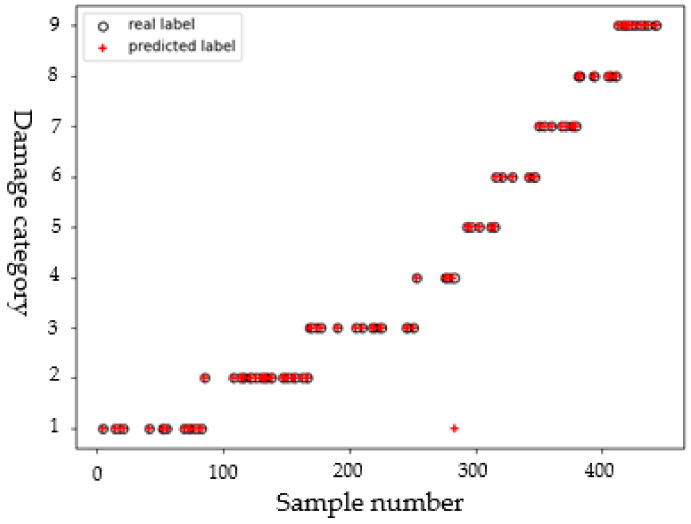
Scatter diagram of identification results of delamination size.

The delamination defects mainly include 9 categories, with a total of 516 samples (6 supplemented samples between different classes are taken as test samples), which are randomly divided into training set and test set in a ratio of 8:2. Referring to the above method, the model kernel function is set as a linear kernel function, the penalty parameter *C* = 2, and the average accuracy rate, recall rate, and $F_{1}$ score are 97.78%, 96.67%, and 97.57%, respectively. Figure 6 is the scatter diagram of the results of identification of delamination size. As can be seen from the figure, only one sample with a size of 5 mm × 8 mm is misclassified into the 5 mm × 5 mm class. The variation in the width of the rectangle has a certain influence on the detection signal. When the sample size is not sufficient to fully cover this variation, it will lead to misclassification of the model.

### 4.3. Parameter Identification Model for Crack

#### 4.3.1. Direction

The scattered field formed by the crack has a certain influence on the guided wave. However, it is difficult to clearly know its law of propagation. In this paper, the LightGBM algorithm is used to identify the crack direction, because it is suitable for high-dimensional data, and its calculation speed is fast. The Hyperparameter Optimization (Hyperopt) method is used to automatically optimize the model parameters.

First, the LightGBM model is used to identify the crack directions on each single path separately, but the effect is not good after parameter adjustment. Taking the path P3–P11 as an example, the average accuracy rate is only 54.29%. This fully shows that in the single detection path, the influence of the ply direction of the composite board and the crack direction cannot be peeled off, which is not conducive to the correct identification of the crack direction by the model.

**Figure 7:**
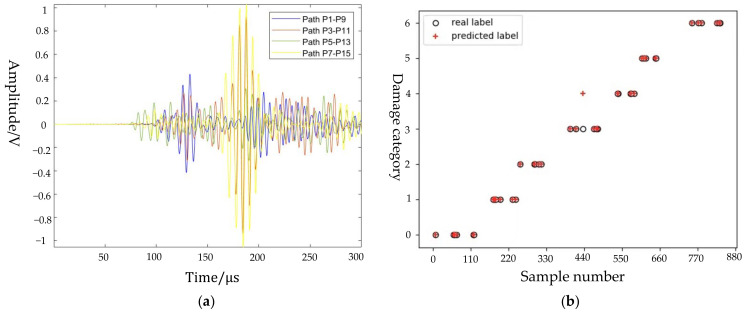
Identification of crack direction: (**a**) comparison of detection signals obtained from different paths for the same defect; and (**b**) scatter diagram of identification results of crack direction.

Comparing the detection signals obtained from different paths for the same defect, as shown in Figure 7a, the distance between the defect and the excitation and receiving point is relatively consistent, resulting in the wave packets of all samples appearing in the time period [100, 300] μs. Therefore, for all defects, the detection samples obtained on the paths P1–P9, P2–P10, P3–P11, P4–P12, P5–P13, P6–P14, P7–P15, and P8–P16 are intercepted and then connected to form new samples with a dimension of 160,000. Dataset with high-dimensional features is randomly divided into training set and test set according to a 9:1 ratio. During parameter adjustment, it is found that the parameters learning_rate and max_depth have a great influence on the classification results. The optimization range of learning_rate is [0.01, 1], the optimization range of max_depth is [1, 15]. Therefore, the optimization result is learning_rate = 0.05, max_depth = 7, and other parameters are set to default. The average accuracy rate, recall rate, and $F_{1}$ score are 97.44%, 98.43%, and 97.71%, respectively.

Figure 7b shows the scatter plot of the classification results after updating the sample size. As can be seen from the figure, only 1 sample in the 60° class is misclassified in the 90° class. This shows that after considering the relationship among the crack direction, the ply direction, and the direction of detection paths to update the dimension of samples, the classification accuracy of the model has been significantly improved.

#### 4.3.2. Length

**Figure 8:**
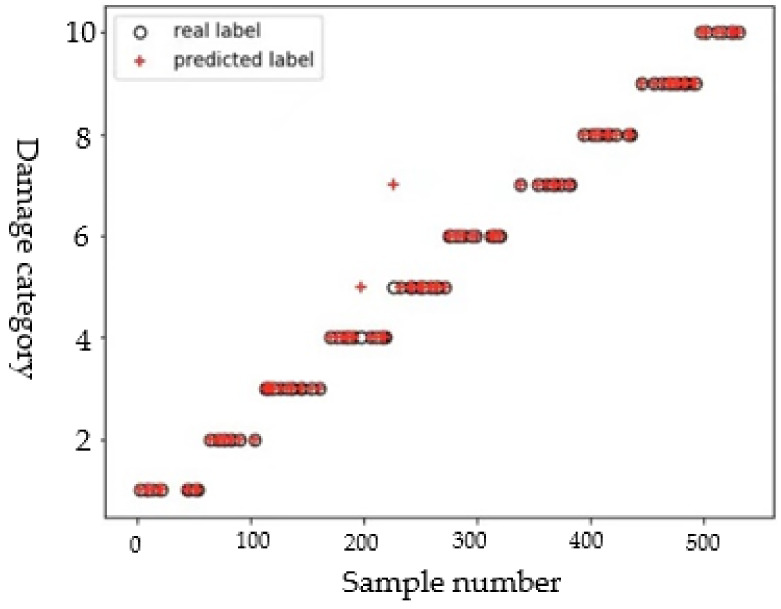
Scatter diagram of crack length.

In each crack direction, when using the detection signals to identify the crack length, these detection signals should be taken from the path perpendicular to the crack direction. For example, the detection signal for a direction of 45° is taken from the path P7–P15; and the detection signal for a direction of 90° is taken from the path P1–P9. The 880 samples obtained in each direction are trained separately to identify the crack length in the corresponding direction. Taking the dataset of crack length in the 45° as an example, the 880 crack samples are randomly divided into a training set and a test set according to an 8:2 ratio. Referring to the method of identifying delamination parameters, the kernel function is set as a linear kernel function, and the penalty parameter *C* = 2. It can be seen from the scatter diagram in Figure 8 that two samples are misclassified to other classes. Since some paths for acquiring detection signals cannot be strictly perpendicular to the crack direction, the deviation in directions sometimes leads to misclassification of some samples (so, a sample with a length of 6.31 mm is misclassified into the 7 mm class; and a sample with a length of 7.07 mm is misclassified into the 9 mm class). However, most samples are classified correctly, indicating that the model has good performance. Based on the identification results of crack length in all directions, the mean of the classification accuracy, recall, and F1 scores of length samples in each crack direction are 96.84%, 97.12%, and 96.52%, respectively.

## 5. Imaging for Testing Defects

### 5.1. Pre-Locating of Defects

**Figure 9:**
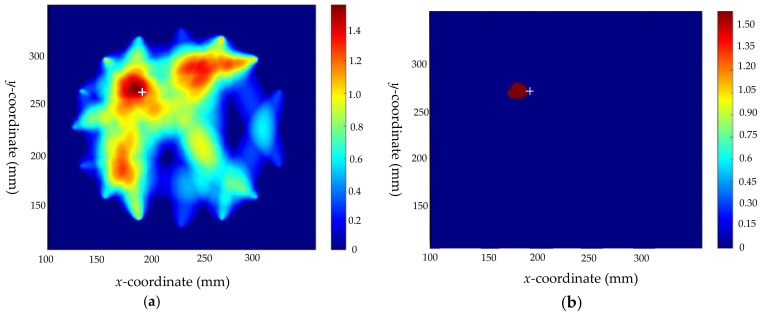
Imaging results before optimization of defect 1: (**a**) before optimization; and (**b**) threshold processing result before optimization.

Taking defect 1 as an example, 18 valid *DI* values are obtained by the above method for reconstructing the image. The image of the defect is reconstructed by RAPID, and the pre-locating imaging result is shown in Figure 9. The defect center is located at (178,266), which is close to the actual position of the defect shown by “+” in the figure. This shows the effectiveness of RAPID for defect location, yet at the same time that the algorithm is not precise enough and cannot give the type and geometric parameters of defects.

### 5.2. Identification of Defect Features

The circular sensor array is repositioned so that its center is located at the predetermined position as described in Section 5.1. Obtaining detection signals for defect feature parameter identification was performed as follows: First, the defect type identification model is used to determine the type of defect, and the identification result shows that defect 1 is a crack. Then, the direction of the crack is determined by the crack direction identification model, and the identification result shows that the direction of defect 1 is 45°. Finally, the length of the crack under the 45° direction is identified by the crack length model, and the identification result shows that the length of defect 1 is 12 mm.

### 5.3. Precise Imaging of Defects

**Figure 10:**
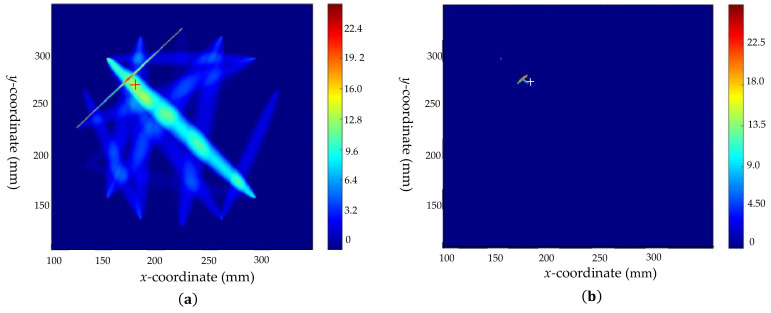
The imaging results of defect 1: (**a**) after optimization; (**b**) threshold processing result after optimization.

Based on the known crack size, the scaling parameter β is calculated according to Equation (4) for paths that are inconsistent with crack direction. Taking paths P7–P15 as an example, the scaling parameter β calculated for this path is 1.004. According to the identification results of the crack direction model, the weight of the cross paths can be calculated by Equation (6) as $W_{i}$ = 17, where the cross paths are P1–P13 (in line with the crack direction) and P7-P15 (perpendicular to the crack direction). The optimized results are shown in Figure 10, where “+” is marked as the real position. Comparing the results of Figure 9 and Figure 10, the optimized results not only reflect the location of the defect, but also intuitively indicate the type, direction, and size of the defects. However, because the crack is extremely narrow in the width direction, and the scaling parameters in this direction are not considered, the obtained localization results still have a certain deviation from the real position.

**Figure 11:**
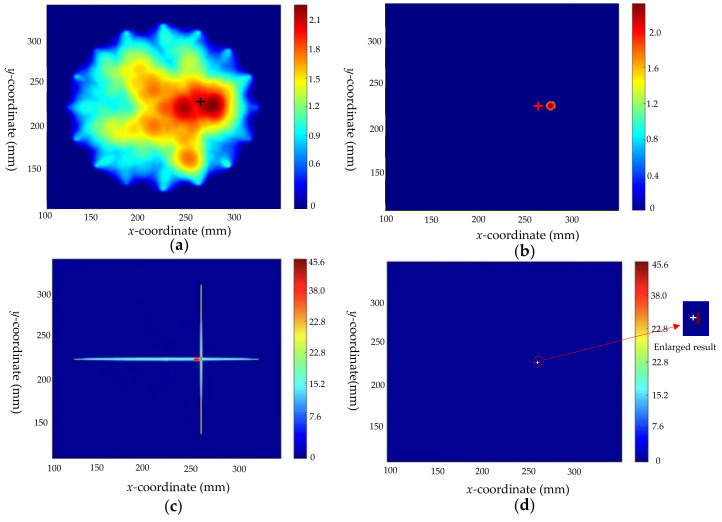
Comparison of imaging results before and after optimization of defect 2: (**a**) imaging results before optimization; (**b**) the threshold processing result before optimization; (**c**) image result after optimization; and (**d**) the threshold processing result after optimization.

**Figure 12:**
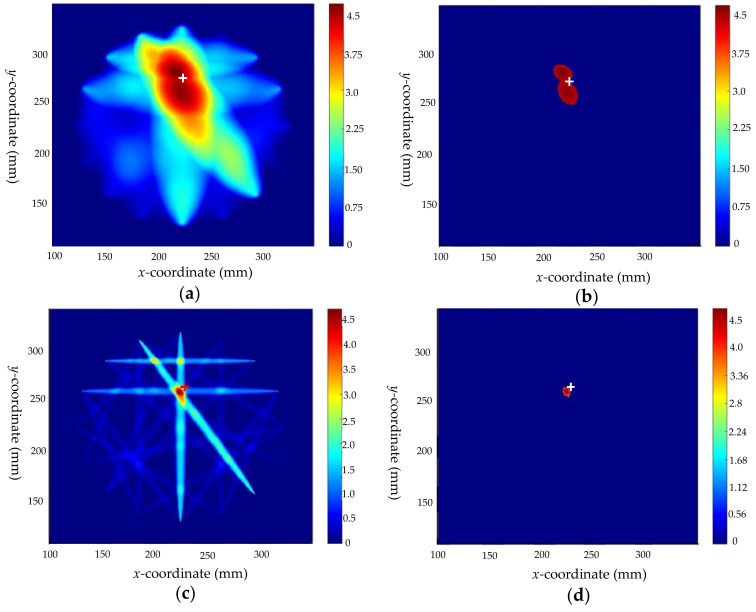
Comparison of imaging results before and after optimization of defect 3: (**a**) imaging results before optimization; (**b**) the threshold processing result before optimization; (**c**) image result after optimization; and (**d**) the threshold processing result after optimization.

Defects 2 and 3 are also studied according to the analysis process of defect 1. Firstly, the RAPID algorithm is used for pre-locating and the results are obtained as shown in Figure 11a and Figure 12a, respectively. After the sensor array position adjustment to regain the required detection signals, the results of crack and delamination are derived from the identification model of defect type, respectively. Next, different feature parameter identification models are applied to predict the crack tilt angle, crack length, and the size of the delamination defect, respectively. The results are shown in Table 4. The scaling parameter β is calculated as mentioned before (taking the paths P1–P9 of defect 2 and P10–P16 of defect 3 as examples, the scaling parameters β are calculated as 1.00053 and 1.0022, respectively) and to set the weights for the “cross paths” (P1–P9 and P6–P12) of defect 2. The coordinate positions of defect centers before and after optimization are shown in Table 4. The comparison plots of the results are shown in Figure 11 and Figure 12, which indicate that the features of the defects are visualized and the imaging positions are closer to the real positions.

## 6. Discussion

In this paper, the proposed method can visually characterize the defects and improve the accuracy of the imaging results. However, there are still some shortcomings in the method.

First, we need to further supplement and improve the sample library; the special case where the size is exactly in the middle of the two classes should be especially considered. For the study of delamination defects, our sample library currently only considers the case when the length and width directions are fixed. In subsequent studies, it will also be necessary to further investigate how to identify the size and direction of delamination when both the length and width directions vary.

Secondly, in relation to the algorithm, the effect of the ellipse factor of the width direction on the imaging results is not considered at present, resulting in the optimized imaging results still having a certain degree of offset compared with the actual position. At the same time, the influence of the neglected path on the imaging results during the path selection process, and whether there is a correspondence between the geometric parameters of the defect and the path selection scheme, need to be further explored in subsequent studies.

Finally, the limited number of sensors in the sensor array can degrade defect imaging quality. The lower the density of the sensor array, the lower the image quality. For oblique cracks, the fewer the sensors arranged in the detection area, the fewer sensing paths there will be. An insufficient number of sensors will result in the inability to select a suitable path to characterize the crack. As described above, the detection signal of the path perpendicular to the crack direction is selected to identify the crack length. When the direction of the selected path deviates from the desired direction, the recognition result of the crack length will be affected. Even when the number of sensors is severely insufficient, this will also lead to the inability to select a suitable path to characterize the crack. Determining how many cells should be contained in the sensor array to be most suitable for the final imaging for defects of different sizes will also need to be further discussed in further research.

## 7. Conclusions

In this paper, an improved RAPID imaging method for composite plates based on machine learning is proposed. The improved method consists of two main parts: (1) Using multiple machine learning models, the main features of defects, including type, size and direction, are identified by analyzing the detection signals. (2) Based on these defect features, the ellipse area scaling parameter β of the RAPID method is modified and weights are set for the detection paths associated with the features to visualize the defect features and effectively improve the accuracy of defect imaging.

(1) Appropriate ML models can be used to predict characteristics of defects such as the type of defect, the size of the delamination, and the length and the direction of the crack.
(2) The RAPID can be used for imaging of composite plates. After the type of defects and main parameter are predicted by the ML models, the *β* value in the algorithm can be further corrected and weights applied to the relevant paths, so that the predicted position is closer to the real position and the geometric characteristics of the defect can be visually displayed in the imaging results.
(3) The relationship between the crack direction, the ply direction, and the angle of detection paths comprehensively affects the detection signal and cannot be easily peeled off from the waveform curve. Therefore, the detection samples obtained under a single detection path are not sufficient to accurately predict the crack direction. Combining the information obtained under multiple detection paths leads to a larger sample dimension, and the combination of different information facilitates the model’s ability to accurately identify the crack direction.

## Figures and Tables

**Table 1:** The acronyms in the defect imaging method.

Symbol	Meaning	Symbol	Meaning
RAPID	The Reconstruction Algorithm for Probabilistic Inspection of Defect	SVM	Support Vector Machine
ML	Machine Learning	PCA	The Principal Component Analysis
DI	Damage Index	C	The Penalty Factor

**Table 2:** Elastic properties of composite plate in the numerical study.

$E_{1}/\mathrm{GPa}$	$E_{2}\;=\;E_{3}/\mathrm{GPa}$	$G_{12}\;=\;G_{13}/\mathrm{GPa}$	$G_{23}/\mathrm{GPa}$	$\upsilon_{12}\;=\;\upsilon_{13}$	$\upsilon_{23}$	$\rho /\left(\mathrm{kg}/m^{3}\right)$
128	82	4.7	3.44	0.27	0.2	1560

**Table 3:** The variation of length and width of delamination between top layer and the adjacent second layer (mm).

Label	Length (mm)	Width (mm)	Number of Samples	Label	Length (mm)	Width (mm)	Number of Samples
1	5, 5.1	5, 5.01, ⋯⋯ 5.2	42	6	10, 10.1	5, 5.01, ⋯⋯ 5.1	22
2	10, 10.1	10, 10.01, ⋯⋯10.2	42	7	10, 10.1	15, 15.01, ⋯⋯ 15.1	22
3	15, 15.1	15, 15.01, ⋯⋯ 15.2	42	8	15, 15.1	5, 5.01, ⋯⋯ 5.1	22
4	5, 5.1	10, 10.01, ⋯⋯10.1	22	9	15, 15.1	10, 10.01, ⋯⋯10.1	22
5	5, 5.1	15, 15.01, ⋯⋯ 15.1	22				

**Table 4:** Comparison of parameters and center coordinates of three defects before and after optimization with the actual situation.

	Actual Defect Parameters and Center Coordinates				Predicted Defect Parameters and Center Coordinates				
	Type	Size (mm)	Direction	Center Coordinates	Type	Size (mm)	Direction	Before Optimization	After Optimization
Defect 1	Crack	12.05	45°	(186,264)	Crack	12	45°	(178,266)	(180,265)
Defect 2	Crack	4.1	90°	(263,225)	Crack	4	90°	(277,227)	(264,225)
Defect 3	Delamination	5 × 5.2	×	(225,263)	Delamination	5 × 5	×	(222,268)	(224,261)

## Data Availability

Not applicable.
